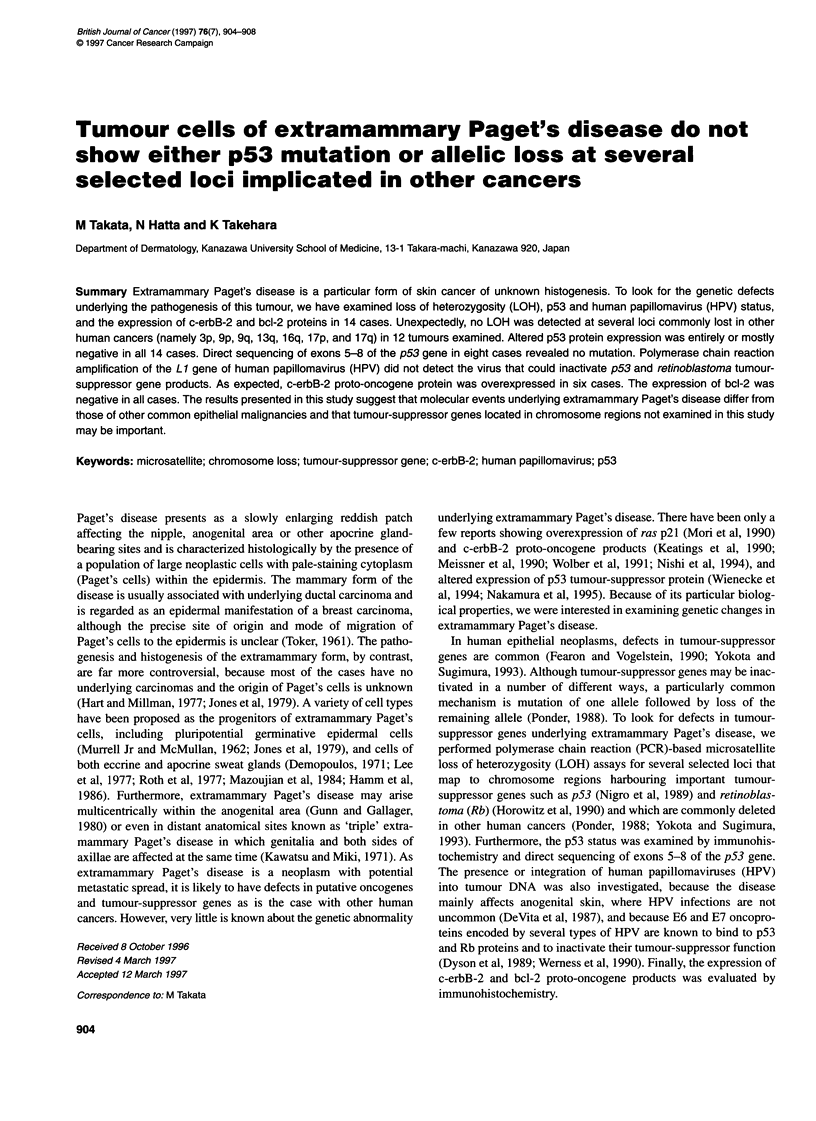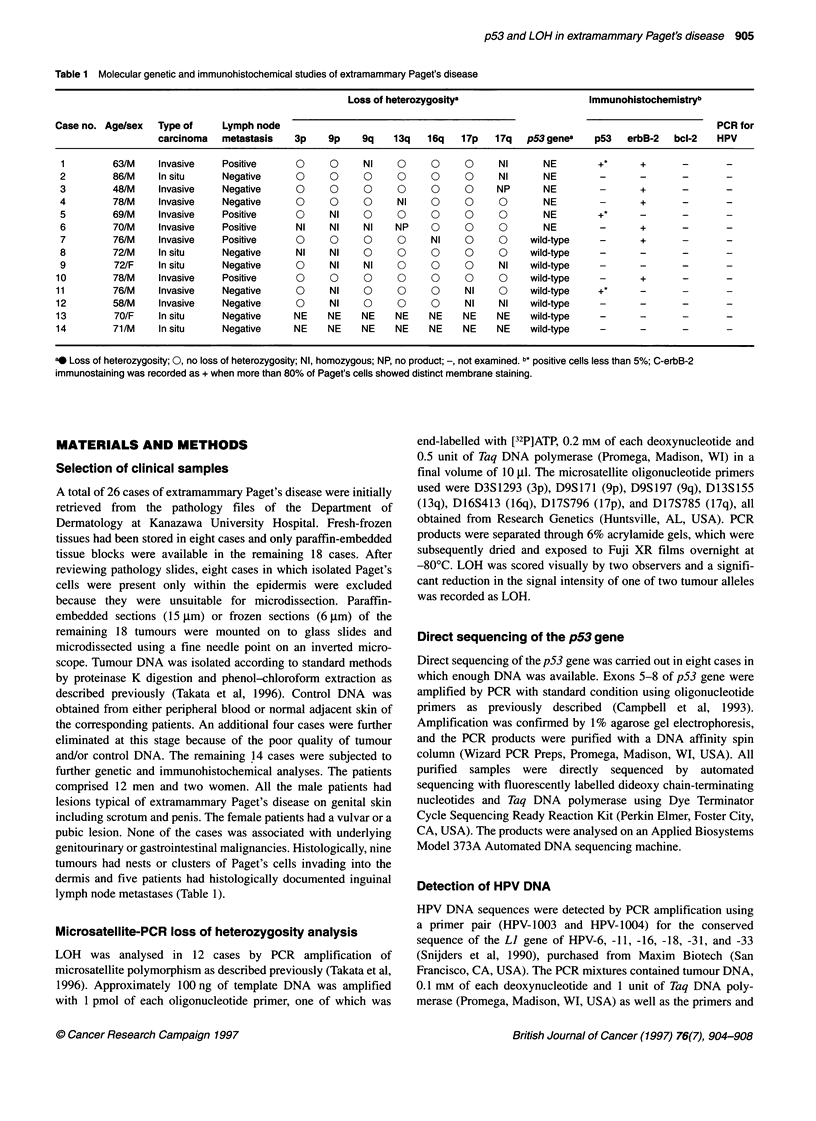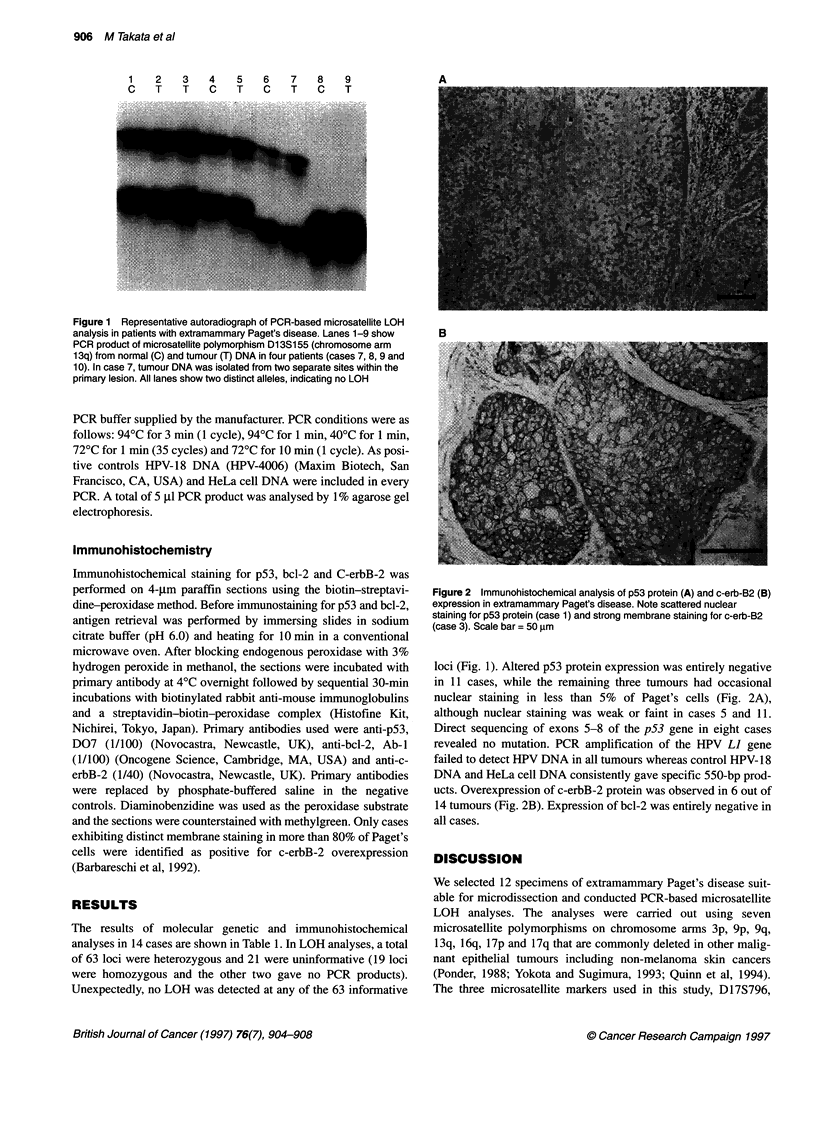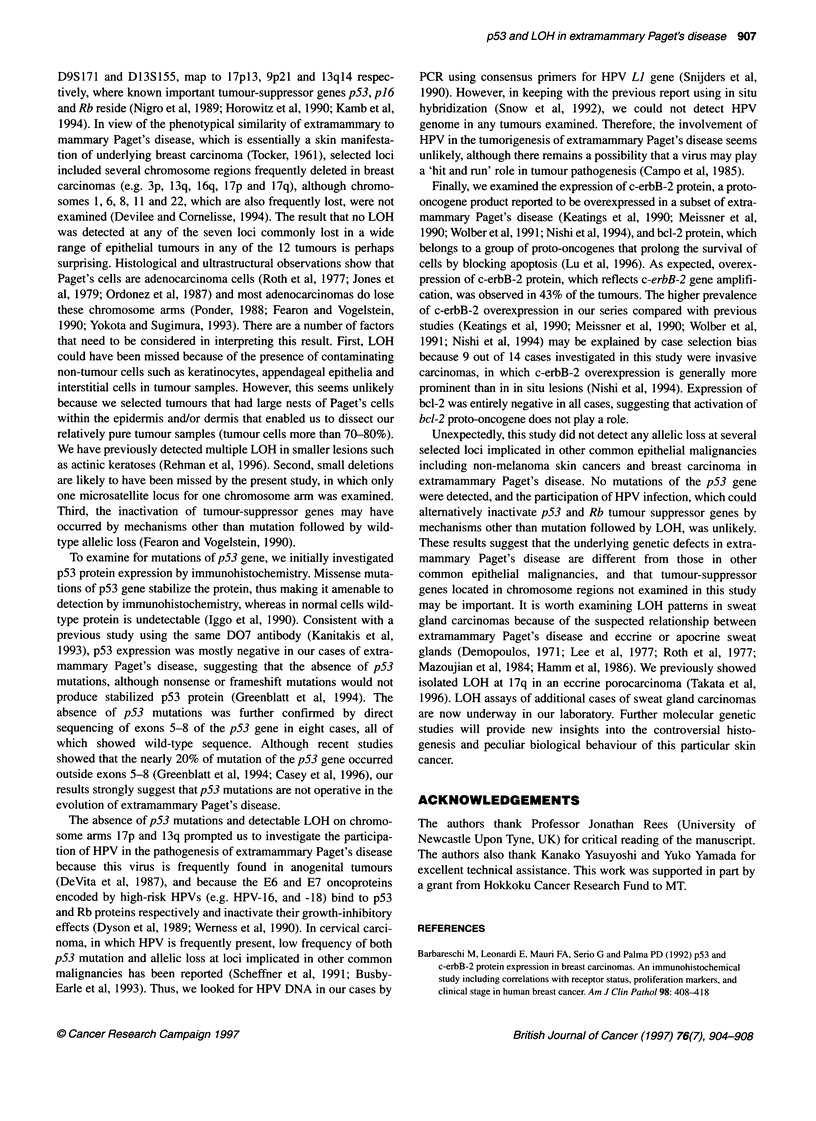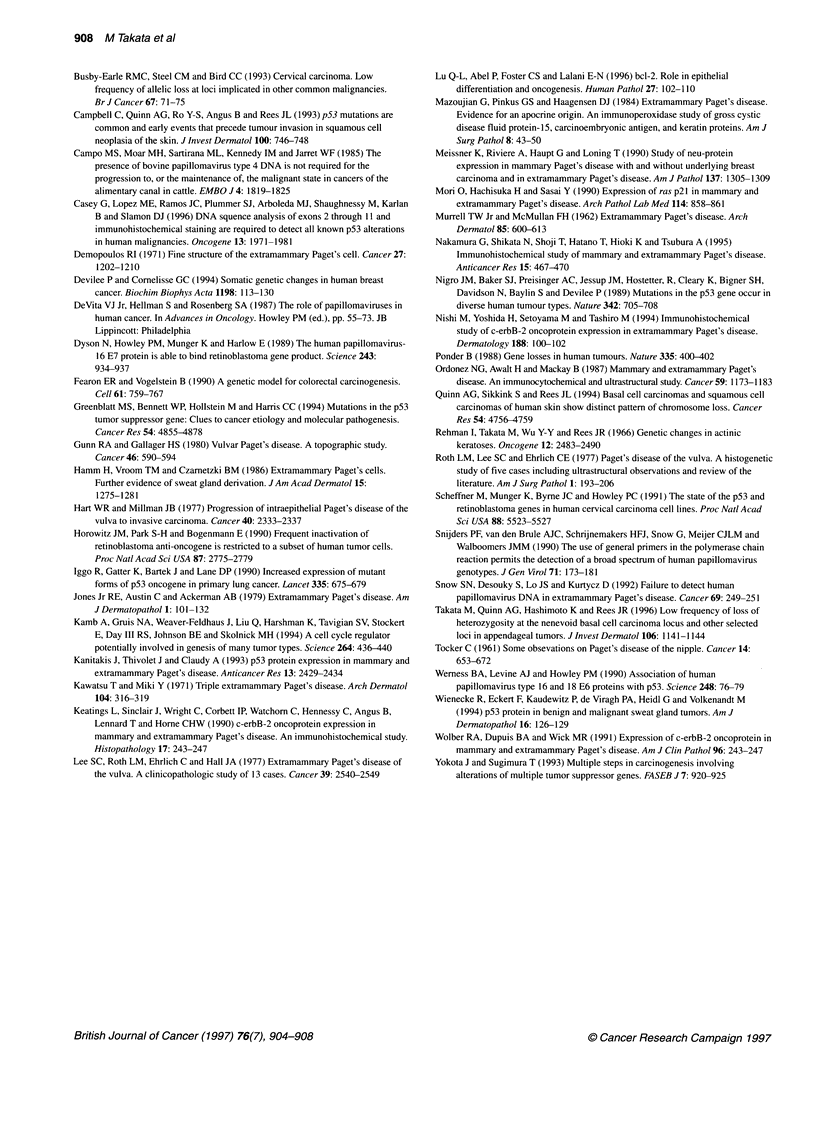# Tumour cells of extramammary Paget's disease do not show either p53 mutation or allelic loss at several selected loci implicated in other cancers.

**DOI:** 10.1038/bjc.1997.482

**Published:** 1997

**Authors:** M. Takata, N. Hatta, K. Takehara

**Affiliations:** Department of Dermatology, Kanazawa University School of Medicine, Japan.

## Abstract

**Images:**


*British Journal of Cancer* (1997) **76**(7), 904–908
© 1997 Cancer Research Campaign

### Tumour cells of extramammary Paget's disease do not show either p53 mutation or allelic loss at several selected loci implicated in other cancers

**M Takata, N Hatta and K Takehara**

Department of Dermatology, Kanazawa University School of Medicine, 13-1 Takara-machi, Kanazawa 920, Japan

**Summary** Extramammary Paget's disease is a particular form of skin cancer of unknown histogenesis. To look for the genetic defects underlying the pathogenesis of this tumour, we have examined loss of heterozygosity (LOH), p53 and human papillomavirus (HPV) status, and the expression of c-erbB-2 and bcl-2 proteins in 14 cases. Unexpectedly, no LOH was detected at several loci commonly lost in other human cancers (namely 3p, 9p, 9q, 13q, 16q, 17p, and 17q) in 12 tumours examined. Altered p53 protein expression was entirely or mostly negative in all 14 cases. Direct sequencing of exons 5–8 of the *p53* gene in eight cases revealed no mutation. Polymerase chain reaction amplification of the *L1* gene of human papillomavirus (HPV) did not detect the virus that could inactivate *p53* and *retinoblastoma* tumour-suppressor gene products. As expected, c-erbB-2 proto-oncogene protein was overexpressed in six cases. The expression of bcl-2 was negative in all cases. The results presented in this study suggest that molecular events underlying extramammary Paget's disease differ from those of other common epithelial malignancies and that tumour-suppressor genes located in chromosome regions not examined in this study may be important.

**Keywords:** microsatellite; chromosome loss; tumour-suppressor gene; c-erbB-2; human papillomavirus; p53

Paget's disease presents as a slowly enlarging reddish patch affecting the nipple, anogenital area or other apocrine gland-bearing sites and is characterized histologically by the presence of a population of large neoplastic cells with pale-staining cytoplasm (Paget's cells) within the epidermis. The mammary form of the disease is usually associated with underlying ductal carcinoma and is regarded as an epidermal manifestation of a breast carcinoma, although the precise site of origin and mode of migration of Paget's cells to the epidermis is unclear (Toker, 1961). The pathogenesis and histogenesis of the extramammary form, by contrast, are far more controversial, because most of the cases have no underlying carcinomas and the origin of Paget's cells is unknown (Hart and Millman, 1977; Jones et al, 1979). A variety of cell types have been proposed as the progenitors of extramammary Paget's cells, including pluripotential germinative epidermal cells (Murrell Jr and McMullan, 1962; Jones et al, 1979), and cells of both eccrine and apocrine sweat glands (Demopoulos, 1971; Lee et al, 1977; Roth et al, 1977; Mazoujian et al, 1984; Hamm et al, 1986). Furthermore, extramammary Paget's disease may arise multicentrically within the anogenital area (Gunn and Gallager, 1980) or even in distant anatomical sites known as 'triple' extramammary Paget's disease in which genitalia and both sides of axillae are affected at the same time (Kawatsu and Miki, 1971). As extramammary Paget's disease is a neoplasm with potential metastatic spread, it is likely to have defects in putative oncogenes and tumour-suppressor genes as is the case with other human cancers. However, very little is known about the genetic abnormality underlying extramammary Paget's disease. There have been only a few reports showing overexpression of *ras* p21 (Mori et al, 1990) and c-erbB-2 proto-oncogene products (Keatings et al, 1990; Meissner et al, 1990; Wolber et al, 1991; Nishi et al, 1994), and altered expression of p53 tumour-suppressor protein (Wienecke et al, 1994; Nakamura et al, 1995). Because of its particular biological properties, we were interested in examining genetic changes in extramammary Paget's disease.

In human epithelial neoplasms, defects in tumour-suppressor genes are common (Fearon and Vogelstein, 1990; Yokota and Sugimura, 1993). Although tumour-suppressor genes may be inactivated in a number of different ways, a particularly common mechanism is mutation of one allele followed by loss of the remaining allele (Ponder, 1988). To look for defects in tumour-suppressor genes underlying extramammary Paget's disease, we performed polymerase chain reaction (PCR)-based microsatellite loss of heterozygosity (LOH) assays for several selected loci that map to chromosome regions harbouring important tumour-suppressor genes such as *p53* (Nigro et al, 1989) and *retinoblastoma* (*Rb*) (Horowitz et al, 1990) and which are commonly deleted in other human cancers (Ponder, 1988; Yokota and Sugimura, 1993). Furthermore, the p53 status was examined by immunohistochemistry and direct sequencing of exons 5–8 of the *p53* gene. The presence or integration of human papillomaviruses (HPV) into tumour DNA was also investigated, because the disease mainly affects anogenital skin, where HPV infections are not uncommon (DeVita et al, 1987), and because E6 and E7 oncoproteins encoded by several types of HPV are known to bind to p53 and Rb proteins and to inactivate their tumour-suppressor function (Dyson et al, 1989; Werness et al, 1990). Finally, the expression of c-erbB-2 and bcl-2 proto-oncogene products was evaluated by immunohistochemistry.

*Received 8 October 1996*
*Revised 4 March 1997*
*Accepted 12 March 1997*

*Correspondence to:* M Takata

**Table 1** Molecular genetic and immunohistochemical studies of extramammary Paget's disease

| Case no. | Age/sex | Type of carcinoma | Lymph node metastasis | Loss of heterozygosity[a] | | | | | | | | Immunohistochemistry[b] | | | PCR for HPV |
|---|---|---|---|---|---|---|---|---|---|---|---|---|---|---|---|
| | | | | 3p | 9p | 9q | 13q | 16q | 17p | 17q | *p53* gene[a] | p53 | erbB-2 | bcl-2 | |
| 1 | 63/M | Invasive | Positive | ○ | ○ | NI | ○ | ○ | ○ | NI | NE | +* | + | – | – |
| 2 | 86/M | In situ | Negative | ○ | ○ | ○ | ○ | ○ | ○ | NI | NE | – | – | – | – |
| 3 | 48/M | Invasive | Negative | ○ | ○ | ○ | ○ | ○ | ○ | NP | NE | – | + | – | – |
| 4 | 78/M | Invasive | Negative | ○ | ○ | ○ | NI | ○ | ○ | ○ | NE | – | + | – | – |
| 5 | 69/M | Invasive | Positive | ○ | NI | ○ | ○ | ○ | ○ | ○ | NE | +* | – | – | – |
| 6 | 70/M | Invasive | Positive | NI | NI | NI | NP | ○ | ○ | ○ | NE | – | + | – | – |
| 7 | 76/M | Invasive | Positive | ○ | ○ | ○ | ○ | NI | ○ | ○ | wild-type | – | + | – | – |
| 8 | 72/M | In situ | Negative | NI | NI | ○ | ○ | ○ | ○ | ○ | wild-type | – | – | – | – |
| 9 | 72/F | In situ | Negative | ○ | NI | NI | ○ | ○ | ○ | NI | wild-type | – | – | – | – |
| 10 | 78/M | Invasive | Positive | ○ | ○ | ○ | ○ | ○ | ○ | ○ | wild-type | – | + | – | – |
| 11 | 76/M | Invasive | Negative | ○ | NI | ○ | ○ | ○ | NI | ○ | wild-type | +* | – | – | – |
| 12 | 58/M | Invasive | Negative | ○ | NI | ○ | ○ | ○ | NI | NI | wild-type | – | – | – | – |
| 13 | 70/F | In situ | Negative | NE | NE | NE | NE | NE | NE | NE | wild-type | – | – | – | – |
| 14 | 71/M | In situ | Negative | NE | NE | NE | NE | NE | NE | NE | wild-type | – | – | – | – |

[a]● Loss of heterozygosity; ○, no loss of heterozygosity; NI, homozygous; NP, no product; –, not examined. [b]* positive cells less than 5%; C-erbB-2 immunostaining was recorded as + when more than 80% of Paget's cells showed distinct membrane staining.

### MATERIALS AND METHODS

### Selection of clinical samples

A total of 26 cases of extramammary Paget's disease were initially retrieved from the pathology files of the Department of Dermatology at Kanazawa University Hospital. Fresh-frozen tissues had been stored in eight cases and only paraffin-embedded tissue blocks were available in the remaining 18 cases. After reviewing pathology slides, eight cases in which isolated Paget's cells were present only within the epidermis were excluded because they were unsuitable for microdissection. Paraffin-embedded sections (15 µm) or frozen sections (6 µm) of the remaining 18 tumours were mounted on to glass slides and microdissected using a fine needle point on an inverted microscope. Tumour DNA was isolated according to standard methods by proteinase K digestion and phenol–chloroform extraction as described previously (Takata et al, 1996). Control DNA was obtained from either peripheral blood or normal adjacent skin of the corresponding patients. An additional four cases were further eliminated at this stage because of the poor quality of tumour and/or control DNA. The remaining 14 cases were subjected to further genetic and immunohistochemical analyses. The patients comprised 12 men and two women. All the male patients had lesions typical of extramammary Paget's disease on genital skin including scrotum and penis. The female patients had a vulvar or a pubic lesion. None of the cases was associated with underlying genitourinary or gastrointestinal malignancies. Histologically, nine tumours had nests or clusters of Paget's cells invading into the dermis and five patients had histologically documented inguinal lymph node metastases (Table 1).

### Microsatellite-PCR loss of heterozygosity analysis

LOH was analysed in 12 cases by PCR amplification of microsatellite polymorphism as described previously (Takata et al, 1996). Approximately 100 ng of template DNA was amplified with 1 pmol of each oligonucleotide primer, one of which was end-labelled with [$^{32}$P]ATP, 0.2 mM of each deoxynucleotide and 0.5 unit of *Taq* DNA polymerase (Promega, Madison, WI) in a final volume of 10 µl. The microsatellite oligonucleotide primers used were D3S1293 (3p), D9S171 (9p), D9S197 (9q), D13S155 (13q), D16S413 (16q), D17S796 (17p), and D17S785 (17q), all obtained from Research Genetics (Huntsville, AL, USA). PCR products were separated through 6% acrylamide gels, which were subsequently dried and exposed to Fuji XR films overnight at –80°C. LOH was scored visually by two observers and a significant reduction in the signal intensity of one of two tumour alleles was recorded as LOH.

### Direct sequencing of the *p53* gene

Direct sequencing of the *p53* gene was carried out in eight cases in which enough DNA was available. Exons 5–8 of *p53* gene were amplified by PCR with standard condition using oligonucleotide primers as previously described (Campbell et al, 1993). Amplification was confirmed by 1% agarose gel electrophoresis, and the PCR products were purified with a DNA affinity spin column (Wizard PCR Preps, Promega, Madison, WI, USA). All purified samples were directly sequenced by automated sequencing with fluorescently labelled dideoxy chain-terminating nucleotides and *Taq* DNA polymerase using Dye Terminator Cycle Sequencing Ready Reaction Kit (Perkin Elmer, Foster City, CA, USA). The products were analysed on an Applied Biosystems Model 373A Automated DNA sequencing machine.

### Detection of HPV DNA

HPV DNA sequences were detected by PCR amplification using a primer pair (HPV-1003 and HPV-1004) for the conserved sequence of the *L1* gene of HPV-6, -11, -16, -18, -31, and -33 (Snijders et al, 1990), purchased from Maxim Biotech (San Francisco, CA, USA). The PCR mixtures contained tumour DNA, 0.1 mM of each deoxynucleotide and 1 unit of *Taq* DNA polymerase (Promega, Madison, WI, USA) as well as the primers and

© Cancer Research Campaign 1997

Figure 1 Representative autoradiograph of PCR-based microsatellite LOH analysis in patients with extramammary Paget's disease. Lanes 1–9 show PCR product of microsatellite polymorphism D13S155 (chromosome arm 13q) from normal (C) and tumour (T) DNA in four patients (cases 7, 8, 9 and 10). In case 7, tumour DNA was isolated from two separate sites within the primary lesion. All lanes show two distinct alleles, indicating no LOH

Figure 2 Immunohistochemical analysis of p53 protein (A) and c-erb-B2 (B) expression in extramammary Paget's disease. Note scattered nuclear staining for p53 protein (case 1) and strong membrane staining for c-erb-B2 (case 3). Scale bar = 50 μm

PCR buffer supplied by the manufacturer. PCR conditions were as follows: 94°C for 3 min (1 cycle), 94°C for 1 min, 40°C for 1 min, 72°C for 1 min (35 cycles) and 72°C for 10 min (1 cycle). As positive controls HPV-18 DNA (HPV-4006) (Maxim Biotech, San Francisco, CA, USA) and HeLa cell DNA were included in every PCR. A total of 5 μl PCR product was analysed by 1% agarose gel electrophoresis.

### Immunohistochemistry

Immunohistochemical staining for p53, bcl-2 and C-erbB-2 was performed on 4-μm paraffin sections using the biotin–streptavidine–peroxidase method. Before immunostaining for p53 and bcl-2, antigen retrieval was performed by immersing slides in sodium citrate buffer (pH 6.0) and heating for 10 min in a conventional microwave oven. After blocking endogenous peroxidase with 3% hydrogen peroxide in methanol, the sections were incubated with primary antibody at 4°C overnight followed by sequential 30-min incubations with biotinylated rabbit anti-mouse immunoglobulins and a streptavidin–biotin–peroxidase complex (Histofine Kit, Nichirei, Tokyo, Japan). Primary antibodies used were anti-p53, DO7 (1/100) (Novocastra, Newcastle, UK), anti-bcl-2, Ab-1 (1/100) (Oncogene Science, Cambridge, MA, USA) and anti-c-erbB-2 (1/40) (Novocastra, Newcastle, UK). Primary antibodies were replaced by phosphate-buffered saline in the negative controls. Diaminobenzidine was used as the peroxidase substrate and the sections were counterstained with methylgreen. Only cases exhibiting distinct membrane staining in more than 80% of Paget's cells were identified as positive for c-erbB-2 overexpression (Barbareschi et al, 1992).

### RESULTS

The results of molecular genetic and immunohistochemical analyses in 14 cases are shown in Table 1. In LOH analyses, a total of 63 loci were heterozygous and 21 were uninformative (19 loci were homozygous and the other two gave no PCR products). Unexpectedly, no LOH was detected at any of the 63 informative loci (Fig. 1). Altered p53 protein expression was entirely negative in 11 cases, while the remaining three tumours had occasional nuclear staining in less than 5% of Paget's cells (Fig. 2A), although nuclear staining was weak or faint in cases 5 and 11. Direct sequencing of exons 5–8 of the *p53* gene in eight cases revealed no mutation. PCR amplification of the HPV *L1* gene failed to detect HPV DNA in all tumours whereas control HPV-18 DNA and HeLa cell DNA consistently gave specific 550-bp products. Overexpression of c-erbB-2 protein was observed in 6 out of 14 tumours (Fig. 2B). Expression of bcl-2 was entirely negative in all cases.

### DISCUSSION

We selected 12 specimens of extramammary Paget's disease suitable for microdissection and conducted PCR-based microsatellite LOH analyses. The analyses were carried out using seven microsatellite polymorphisms on chromosome arms 3p, 9p, 9q, 13q, 16q, 17p and 17q that are commonly deleted in other malignant epithelial tumours including non-melanoma skin cancers (Ponder, 1988; Yokota and Sugimura, 1993; Quinn et al, 1994). The three microsatellite markers used in this study, D17S796,

 © Cancer Research Campaign 1997

D9S171 and D13S155, map to 17p13, 9p21 and 13q14 respectively, where known important tumour-suppressor genes *p53*, *p16* and *Rb* reside (Nigro et al, 1989; Horowitz et al, 1990; Kamb et al, 1994). In view of the phenotypical similarity of extramammary to mammary Paget's disease, which is essentially a skin manifestation of underlying breast carcinoma (Tocker, 1961), selected loci included several chromosome regions frequently deleted in breast carcinomas (e.g. 3p, 13q, 16q, 17p and 17q), although chromosomes 1, 6, 8, 11 and 22, which are also frequently lost, were not examined (Devilee and Cornelisse, 1994). The result that no LOH was detected at any of the seven loci commonly lost in a wide range of epithelial tumours in any of the 12 tumours is perhaps surprising. Histological and ultrastructural observations show that Paget's cells are adenocarcinoma cells (Roth et al, 1977; Jones et al, 1979; Ordonez et al, 1987) and most adenocarcinomas do lose these chromosome arms (Ponder, 1988; Fearon and Vogelstein, 1990; Yokota and Sugimura, 1993). There are a number of factors that need to be considered in interpreting this result. First, LOH could have been missed because of the presence of contaminating non-tumour cells such as keratinocytes, appendageal epithelia and interstitial cells in tumour samples. However, this seems unlikely because we selected tumours that had large nests of Paget's cells within the epidermis and/or dermis that enabled us to dissect our relatively pure tumour samples (tumour cells more than 70–80%). We have previously detected multiple LOH in smaller lesions such as actinic keratoses (Rehman et al, 1996). Second, small deletions are likely to have been missed by the present study, in which only one microsatellite locus for one chromosome arm was examined. Third, the inactivation of tumour-suppressor genes may have occurred by mechanisms other than mutation followed by wild-type allelic loss (Fearon and Vogelstein, 1990).

To examine for mutations of *p53* gene, we initially investigated p53 protein expression by immunohistochemistry. Missense mutations of p53 gene stabilize the protein, thus making it amenable to detection by immunohistochemistry, whereas in normal cells wild-type protein is undetectable (Iggo et al, 1990). Consistent with a previous study using the same DO7 antibody (Kanitakis et al, 1993), p53 expression was mostly negative in our cases of extramammary Paget's disease, suggesting that the absence of *p53* mutations, although nonsense or frameshift mutations would not produce stabilized p53 protein (Greenblatt et al, 1994). The absence of *p53* mutations was further confirmed by direct sequencing of exons 5–8 of the *p53* gene in eight cases, all of which showed wild-type sequence. Although recent studies showed that the nearly 20% of mutation of the *p53* gene occurred outside exons 5–8 (Greenblatt et al, 1994; Casey et al, 1996), our results strongly suggest that *p53* mutations are not operative in the evolution of extramammary Paget's disease.

The absence of *p53* mutations and detectable LOH on chromosome arms 17p and 13q prompted us to investigate the participation of HPV in the pathogenesis of extramammary Paget's disease because this virus is frequently found in anogenital tumours (DeVita et al, 1987), and because the E6 and E7 oncoproteins encoded by high-risk HPVs (e.g. HPV-16, and -18) bind to p53 and Rb proteins respectively and inactivate their growth-inhibitory effects (Dyson et al, 1989; Werness et al, 1990). In cervical carcinoma, in which HPV is frequently present, low frequency of both *p53* mutation and allelic loss at loci implicated in other common malignancies has been reported (Scheffner et al, 1991; Busby-Earle et al, 1993). Thus, we looked for HPV DNA in our cases by PCR using consensus primers for HPV *L1* gene (Snijders et al, 1990). However, in keeping with the previous report using in situ hybridization (Snow et al, 1992), we could not detect HPV genome in any tumours examined. Therefore, the involvement of HPV in the tumorigenesis of extramammary Paget's disease seems unlikely, although there remains a possibility that a virus may play a 'hit and run' role in tumour pathogenesis (Campo et al, 1985).

Finally, we examined the expression of c-erbB-2 protein, a proto-oncogene product reported to be overexpressed in a subset of extramammary Paget's disease (Keatings et al, 1990; Meissner et al, 1990; Wolber et al, 1991; Nishi et al, 1994), and bcl-2 protein, which belongs to a group of proto-oncogenes that prolong the survival of cells by blocking apoptosis (Lu et al, 1996). As expected, overexpression of c-erbB-2 protein, which reflects *c-erbB-2* gene amplification, was observed in 43% of the tumours. The higher prevalence of c-erbB-2 overexpression in our series compared with previous studies (Keatings et al, 1990; Meissner et al, 1990; Wolber et al, 1991; Nishi et al, 1994) may be explained by case selection bias because 9 out of 14 cases investigated in this study were invasive carcinomas, in which c-erbB-2 overexpression is generally more prominent than in in situ lesions (Nishi et al, 1994). Expression of bcl-2 was entirely negative in all cases, suggesting that activation of *bcl-2* proto-oncogene does not play a role.

Unexpectedly, this study did not detect any allelic loss at several selected loci implicated in other common epithelial malignancies including non-melanoma skin cancers and breast carcinoma in extramammary Paget's disease. No mutations of the *p53* gene were detected, and the participation of HPV infection, which could alternatively inactivate *p53* and *Rb* tumour suppressor genes by mechanisms other than mutation followed by LOH, was unlikely. These results suggest that the underlying genetic defects in extramammary Paget's disease are different from those in other common epithelial malignancies, and that tumour-suppressor genes located in chromosome regions not examined in this study may be important. It is worth examining LOH patterns in sweat gland carcinomas because of the suspected relationship between extramammary Paget's disease and eccrine or apocrine sweat glands (Demopoulos, 1971; Lee et al, 1977; Roth et al, 1977; Mazoujian et al, 1984; Hamm et al, 1986). We previously showed isolated LOH at 17q in an eccrine porocarcinoma (Takata et al, 1996). LOH assays of additional cases of sweat gland carcinomas are now underway in our laboratory. Further molecular genetic studies will provide new insights into the controversial histogenesis and peculiar biological behaviour of this particular skin cancer.

### ACKNOWLEDGEMENTS

The authors thank Professor Jonathan Rees (University of Newcastle Upon Tyne, UK) for critical reading of the manuscript. The authors also thank Kanako Yasuyoshi and Yuko Yamada for excellent technical assistance. This work was supported in part by a grant from Hokkoku Cancer Research Fund to MT.

### REFERENCES

Barbareschi M, Leonardi E, Mauri FA, Serio G and Palma PD (1992) p53 and c-erbB-2 protein expression in breast carcinomas. An immunohistochemical study including correlations with receptor status, proliferation markers, and clinical stage in human breast cancer. *Am J Clin Pathol* **98**: 408–418

© Cancer Research Campaign 1997

Busby-Earle RMC, Steel CM and Bird CC (1993) Cervical carcinoma. Low frequency of allelic loss at loci implicated in other common malignancies. *Br J Cancer* **67**: 71–75

Campbell C, Quinn AG, Ro Y-S, Angus B and Rees JL (1993) *p53* mutations are common and early events that precede tumour invasion in squamous cell neoplasia of the skin. *J Invest Dermatol* **100**: 746–748

Campo MS, Moar MH, Sartirana ML, Kennedy IM and Jarret WF (1985) The presence of bovine papillomavirus type 4 DNA is not required for the progression to, or the maintenance of, the malignant state in cancers of the alimentary canal in cattle. *EMBO J* **4**: 1819–1825

Casey G, Lopez ME, Ramos JC, Plummer SJ, Arboleda MJ, Shaughnessy M, Karlan B and Slamon DJ (1996) DNA squence analysis of exons 2 through 11 and immunohistochemical staining are required to detect all known p53 alterations in human malignancies. *Oncogene* **13**: 1971–1981

Demopoulos RI (1971) Fine structure of the extramammary Paget's cell. *Cancer* **27**: 1202–1210

Devilee P and Cornelisse GC (1994) Somatic genetic changes in human breast cancer. *Biochim Biophys Acta* **1198**: 113–130

DeVita VJ Jr, Hellman S and Rosenberg SA (1987) The role of papillomaviruses in human cancer. In *Advances in Oncology*. Howley PM (ed.), pp. 55–73. JB Lippincott: Philadelphia

Dyson N, Howley PM, Munger K and Harlow E (1989) The human papillomavirus-16 E7 protein is able to bind retinoblastoma gene product. *Science* **243**: 934–937

Fearon ER and Vogelstein B (1990) A genetic model for colorectal carcinogenesis. *Cell* **61**: 759–767

Greenblatt MS, Bennett WP, Hollstein M and Harris CC (1994) Mutations in the p53 tumor suppressor gene: Clues to cancer etiology and molecular pathogenesis. *Cancer Res* **54**: 4855–4878

Gunn RA and Gallager HS (1980) Vulvar Paget's disease. A topographic study. *Cancer* **46**: 590–594

Hamm H, Vroom TM and Czarnetzki BM (1986) Extramammary Paget's cells. Further evidence of sweat gland derivation. *J Am Acad Dermatol* **15**: 1275–1281

Hart WR and Millman JB (1977) Progression of intraepithelial Paget's disease of the vulva to invasive carcinoma. *Cancer* **40**: 2333–2337

Horowitz JM, Park S-H and Bogenmann E (1990) Frequent inactivation of retinoblastoma anti-oncogene is restricted to a subset of human tumor cells. *Proc Natl Acad Sci USA* **87**: 2775–2779

Iggo R, Gatter K, Bartek J and Lane DP (1990) Increased expression of mutant forms of p53 oncogene in primary lung cancer. *Lancet* **335**: 675–679

Jones Jr RE, Austin C and Ackerman AB (1979) Extramammary Paget's disease. *Am J Dermatopathol* **1**: 101–132

Kamb A, Gruis NA, Weaver-Feldhaus J, Liu Q, Harshman K, Tavigian SV, Stockert E, Day III RS, Johnson BE and Skolnick MH (1994) A cell cycle regulator potentially involved in genesis of many tumor types. *Science* **264**: 436–440

Kanitakis J, Thivolet J and Claudy A (1993) p53 protein expression in mammary and extramammary Paget's disease. *Anticancer Res* **13**: 2429–2434

Kawatsu T and Miki Y (1971) Triple extramammary Paget's disease. *Arch Dermatol* **104**: 316–319

Keatings L, Sinclair J, Wright C, Corbett IP, Watchorn C, Hennessy C, Angus B, Lennard T and Horne CHW (1990) c-erbB-2 oncoprotein expression in mammary and extramammary Paget's disease. An immunohistochemical study. *Histopathology* **17**: 243–247

Lee SC, Roth LM, Ehrlich C and Hall JA (1977) Extramammary Paget's disease of the vulva. A clinicopathologic study of 13 cases. *Cancer* **39**: 2540–2549

Lu Q-L, Abel P, Foster CS and Lalani E-N (1996) bcl-2. Role in epithelial differentiation and oncogenesis. *Human Pathol* **27**: 102–110

Mazoujian G, Pinkus GS and Haagensen DJ (1984) Extramammary Paget's disease. Evidence for an apocrine origin. An immunoperoxidase study of gross cystic disease fluid protein-15, carcinoembryonic antigen, and keratin proteins. *Am J Surg Pathol* **8**: 43–50

Meissner K, Riviere A, Haupt G and Loning T (1990) Study of neu-protein expression in mammary Paget's disease with and without underlying breast carcinoma and in extramammary Paget's disease. *Am J Pathol* **137**: 1305–1309

Mori O, Hachisuka H and Sasai Y (1990) Expression of *ras* p21 in mammary and extramammary Paget's disease. *Arch Pathol Lab Med* **114**: 858–861

Murrell TW Jr and McMullan FH (1962) Extramammary Paget's disease. *Arch Dermatol* **85**: 600–613

Nakamura G, Shikata N, Shoji T, Hatano T, Hioki K and Tsubura A (1995) Immunohistochemical study of mammary and extramammary Paget's disease. *Anticancer Res* **15**: 467–470

Nigro JM, Baker SJ, Preisinger AC, Jessup JM, Hostetter, R, Cleary K, Bigner SH, Davidson N, Baylin S and Devilee P (1989) Mutations in the p53 gene occur in diverse human tumour types. *Nature* **342**: 705–708

Nishi M, Yoshida H, Setoyama M and Tashiro M (1994) Immunohistochemical study of c-erbB-2 oncoprotein expression in extramammary Paget's disease. *Dermatology* **188**: 100–102

Ponder B (1988) Gene losses in human tumours. *Nature* **335**: 400–402

Ordonez NG, Awalt H and Mackay B (1987) Mammary and extramammary Paget's disease. An immunocytochemical and ultrastructural study. *Cancer* **59**: 1173–1183

Quinn AG, Sikkink S and Rees JL (1994) Basal cell carcinomas and squamous cell carcinomas of human skin show distinct pattern of chromosome loss. *Cancer Res* **54**: 4756–4759

Rehman I, Takata M, Wu Y-Y and Rees JR (1966) Genetic changes in actinic keratoses. *Oncogene* **12**: 2483–2490

Roth LM, Lee SC and Ehrlich CE (1977) Paget's disease of the vulva. A histogenetic study of five cases including ultrastructural observations and review of the literature. *Am J Surg Pathol* **1**: 193–206

Scheffner M, Munger K, Byrne JC and Howley PC (1991) The state of the p53 and retinoblastoma genes in human cervical carcinoma cell lines. *Proc Natl Acad Sci USA* **88**: 5523–5527

Snijders PF, van den Brule AJC, Schrijnemakers HFJ, Snow G, Meijer CJLM and Walboomers JMM (1990) The use of general primers in the polymerase chain reaction permits the detection of a broad spectrum of human papillomavirus genotypes. *J Gen Virol* **71**: 173–181

Snow SN, Desouky S, Lo JS and Kurtycz D (1992) Failure to detect human papillomavirus DNA in extramammary Paget's disease. *Cancer* **69**: 249–251

Takata M, Quinn AG, Hashimoto K and Rees JR (1996) Low frequency of loss of heterozygosity at the nenevoid basal cell carcinoma locus and other selected loci in appendageal tumors. *J Invest Dermatol* **106**: 1141–1144

Tocker C (1961) Some obsevations on Paget's disease of the nipple. *Cancer* **14**: 653–672

Werness BA, Levine AJ and Howley PM (1990) Association of human papillomavirus type 16 and 18 E6 proteins with p53. *Science* **248**: 76–79

Wienecke R, Eckert F, Kaudewitz P, de Viragh PA, Heidl G and Volkenandt M (1994) p53 protein in benign and malignant sweat gland tumors. *Am J Dermatopathol* **16**: 126–129

Wolber RA, Dupuis BA and Wick MR (1991) Expression of c-erbB-2 oncoprotein in mammary and extramammary Paget's disease. *Am J Clin Pathol* **96**: 243–247

Yokota J and Sugimura T (1993) Multiple steps in carcinogenesis involving alterations of multiple tumor suppressor genes. *FASEB J* **7**: 920–925

© Cancer Research Campaign 1997